# Effects of Mindfulness Practice on Owner-Reported Dog Behavior and Relationship

**DOI:** 10.3390/ani16040542

**Published:** 2026-02-10

**Authors:** Claudia Pinelli, Anna Scandurra, Alfredo Di Lucrezia, Angelo Vaira, Emanuela Regazzi, Biagio D’Aniello

**Affiliations:** 1 Department of Environmental, Biological and Pharmaceutical Sciences & Technologies, University of Campania Luigi Vanvitelli, 81100 Caserta, Italy; claudia.pinelli@unicampania.it; 2 Department of Biology, University of Naples Federico II, 80126 Naples, Italy; anna.scandurra@unina.it (A.S.); alfredo.dilucrezia@unina.it (A.D.L.); 3 Vajra Vision Srl, Viale Francesco Restelli 3, 20124 Milan, Italy; a.vaira@thinkdog.it (A.V.); e.regazzi@thinkdog.it (E.R.)

**Keywords:** C-BARQ, MDORS, mindfulness, dog, dog behavior, dog–owner relationship

## Abstract

Mindfulness practices have been shown to improve human emotional regulation and attentional accuracy, yet their impact on the perception of companion animal behavior remains largely unexplored. This study investigates whether a mindfulness program for owners influences their evaluation of dog behavior and the dog–owner relationship. Participants were assigned to either an 8-week Mindfulness-Based Human–Animal Relationship (MBHAR) program (n = 102) or a control group (n = 51). The MBHAR protocol included formal meditation and informal daily exercises practiced in the presence of the dog. Evaluation was conducted at baseline (T0) and post-intervention (T1) using the Italian validated versions of the Canine Behavioral Assessment and Research Questionnaire (C-BARQ) and the Monash Dog–Owner Relationship Scale (MDORS). Data were analyzed using Wilcoxon signed-rank tests and Generalized Linear Mixed Models (GLMMs) to control for age and sex. While initial non-parametric analyses suggested improvements across several C-BARQ factors, after Bonferroni correction and GLMM validation, only “Stranger-directed aggression/fear” showed a robust and significant decrease in owner-reported scores in the mindfulness group compared to the control group. No significant changes were found in MDORS scores, indicating that the perceived quality of the relationship remained stable. The results suggest that mindfulness practice specifically influences how owners perceive and report their dog’s fearful or aggressive responses toward strangers. These findings highlight the potential of mindfulness as a tool to modify owner perception, which may influence the management of canine behavioral challenges, although its impact on the structural dimensions of the dog–owner bond appears limited in the short term.

## Simple Summary

This study investigated whether a mental awareness practice, known as mindfulness, could influence how owners perceive their dogs’ behavior and the quality of their relationship. Mindfulness involves training the mind to focus on the present moment with a calm and non-judgmental attitude. We compared a group of owners who completed an 8-week mindfulness program with a control group. Participants filled out validated surveys providing information about their dog’s behavior both before and after the program. The results showed that owners who practiced mindfulness reported a significant decrease in their dogs’ fear and aggression toward strangers compared to the control group. Other behaviors and the overall quality of the relationship remained stable. These findings suggest that mindfulness can help owners perceive certain difficult dog behaviors more positively or respond to them with greater emotional regulation.

## 1. Introduction

Mindfulness, originally rooted in spiritual and theoretical Buddhist traditions, has evolved into a more practical tool in modern Western settings. While it still incorporates core Buddhist principles, such as present-moment awareness and the development of insight [1], it has also emerged as a distinct cultural phenomenon that differs from traditional practices in specific contexts [2]. Today, mindfulness is generally defined as the intentional, nonjudgmental awareness of the continuous flow of internal and external stimuli as they occur [3,4]. In line with these principles, mindfulness-based meditation encourages individuals to observe their sensations, emotions, and thoughts. Practitioners learn to identify their thoughts without judgment, reduce emotional reactivity, and focus on the present moment rather than past or future events [5]. Different versions of these programs aim to improve mental health, with some focused mainly on stress reduction [6], others on cognitive therapy [7], and others on body scan meditation to enhance body awareness and overall well-being [8]. These interventions emphasize awareness marked by attention regulation towards current experiences, combined with an attitude of acceptance and non-judgment [9]. These programs are delivered through structured formats, ranging from group-based sessions and personal training to increasingly common online and digital platforms, allowing for flexibility based on the participants’ needs.

The popularity of mindfulness has surged, supported by numerous studies confirming its effectiveness in promoting mental health and reducing anxiety, stress, and depression [10,11,12,13,14]. It is also known to improve cognitive functions like reaction time and attention [15], and to increase self-acceptance while reducing negative emotions and self-criticism [8]. Furthermore, it has been demonstrated that mindfulness can alleviate physiological stress, as evidenced by reductions in cortisol levels [16]. Nevertheless, the effectiveness of mindfulness programs is frequently debated; some studies indicate that short-term duration interventions (e.g., two to six months) yield modest improvements in anxiety and depression, comparable to the gains observed with pharmacological antidepressant treatments [17].

Similarly to mindfulness, interactions with companion animals can promote psychological well-being by helping owners focus on the present moment [18]. Dogs offer numerous benefits to human health and well-being, including psychological advantages [18,19,20], and act as a lubricant in social interactions [21], thereby influencing a wide range of human social behaviors [22]. One study explored whether combining dog-assisted therapy with mindfulness could amplify these positive effects, but no significant improvements were found [23]. Similarly, studies on mindfulness combined with the presence of a dog in virtual reality settings showed mixed or modest results [13,24,25,26]. However, the physical presence of a dog during mindful practice has been shown to positively impact owners’ well-being by increasing affiliative and synchronization behaviors [27]. These findings support the concept of a strong connection between the owner’s psychological state and the observed human–animal interaction dynamics. This interdependence is further supported by the established literature on emotional and physiological contagion between owners and their dogs [28], which suggests that shifts in an owner’s internal state can directly influence the dog’s arousal and behavior within the dyad. Beyond direct behavioral influence, an owner’s psychological well-being is known to act as a lens through which they interpret their pet’s actions. For instance, research has shown that owners who report behavioral problems in their dogs, such as separation-related issues, often show higher levels of personal stress and lower happiness compared to those who view their pet as well-behaved [29]. This suggests that a person’s internal distress may contribute to a less relaxed relationship and a more negative appraisal of their dog’s behavior. Given that mindfulness practice is a validated method to reduce perceived stress and modulate social perception, it is plausible that such training could refine how owners interpret and report their dog’s actions, potentially leading to a more balanced evaluation of the pet.

Despite evidence that mindfulness affects human attention and emotional processing, no research to date has explicitly examined how owner participation in mindfulness programs affects the perception of canine behavior. The current study aimed to fill this gap by investigating whether the psychological and attentional benefits associated with mindfulness practice may influence how owners evaluate their dog’s behaviors and the owner–dog relationship, as measured through the validated questionnaires C-BARQ and MDORS. As previously outlined, mindfulness interventions are known to enhance present-moment awareness, emotional regulation, and attentional accuracy in humans. When applied to everyday interactions with dogs, these mechanisms may lead owners to attend more closely to their dog’s communicative cues, interpret them differently, or perceive the dyadic relationship more positively. This interpretation aligns with evidence showing that owners’ psychological traits and emotional well-being influence how they perceive and report their dogs’ behavioral tendencies [30]. Consequently, we hypothesized that owners participating in the MBHAR program would report positive shifts in their perception of their dog’s behavior and in the perceived quality of their relationship.

## 2. Materials and Methods

### 2.1. Participants and Study Design

The study followed a non-randomized, quasi-experimental design, as group assignment was based on the participants’ voluntary enrollment in the mindfulness training course following informational sessions. Participants were drawn from a cohort of individuals interested in mindfulness who had attended preliminary informational sessions prior to the start of the program periodically organized by one of the authors of this study. Those who opted for the mindfulness training were assigned to the mindfulness condition, while those who did not participate at that time but had expressed an equivalent initial interest in the informational sessions, were included in the control condition. All participants resided in Italy. The final sample was predominantly composed of women (C-BARQ = 96%; MDORS = 95%). The dogs involved included both purebred and mixed-breed individuals (purebred C-BARQ = 49%; MDORS = 57%), with an age range from 1 to 12 years for both C-BARQ and MDORS samples. No further restrictions were applied regarding the dogs’ breed or specific age.

The questionnaires were administered online to all volunteers at the beginning of the mindfulness course (T0) and again at the end of the 8-week program (T1) for both groups. To ensure consistency, the 8-week interval between T0 and T1 was maintained for the control group as well. Before conducting the statistical analysis, a series of preliminary procedures were performed to verify data quality and prepare the final dataset (see below).

### 2.2. Mindfulness Description

Nature-based mindfulness integrates natural environments into meditative practices [31], highlighting the connection between individuals and nature as a key therapeutic component for mental health and psychological recovery [32,33,34]. Animal-assisted practices are nature-based mindfulness interventions centered on forming meaningful relationships between humans and animals. The primary method involves developing a mindful connection through techniques like body scanning, aimed at enhancing sensory awareness, practiced in the presence of animals to help individuals stay present, increase self-awareness, and foster acceptance of the present experience [35]. These techniques aim to enhance awareness of behaviors and reactions, positively influencing interpersonal relationships [36].

For the present study, the Mindfulness-Based Human–Animal Relationship (MBHAR) protocol was applied. Developed by one of the authors (A.V.), a certified dog trainer and AIM (Italian Mindfulness Association) instructor, the MBHAR protocol differs from standard Mindfulness-Based Stress Reduction (MBSR) due to the inclusion of animals in meditation sessions. The sessions were conducted via online video calls. During these sessions, the instructor provided verbal guidance and demonstrated the techniques without the presence of a dog. Only the participants were at home with their dogs, who were free to interact with them in the same room throughout the practice. Specifically, the animal is free to approach the practitioner, who is encouraged to include the presence of the animal and any interactions with it in their field of awareness. The protocol is structured over 8 weeks and delivered via synchronous online sessions (Zoom Video Communications, Inc., San Jose, CA, USA). The intervention combines three main components: (i) Formal Practice: dedicated time for structured meditation, performed daily at home for 45 min guided by audio recordings provided by the instructor; (ii) Informal Practice: exercises of variable duration designed to bring mindful awareness into daily activities. This includes specially designed exercises to be performed with the animal, such as sharing food, mindful walking, opening to sounds and smells, etc. For instance, participants were tasked with 10–15 min of mindful grooming, where they focused on the tactile sensations and the animal’s responses, or the conscious observation of their dog’s breathing patterns during rest to deepen their attunement to the animal’s physiological state; (iii) Weekly Group Sessions: participants met with the instructor once a week for 2.5 h via Zoom. These meetings included guided group meditations and inquiry periods, structured moments of reflection where participants share and elaborate on their experiences with the group. Under the guidance of the instructor, participants engaged in a non-judgmental exploration of their experiences, maintaining a continuous focus on description alone and deliberately setting aside interpretation and narrative.

### 2.3. C-BARQ and MDORS Assessment

In this study, a methodological approach based on owner-reported questionnaires was adopted. For data collection, the Canine Behavioral Assessment and Research Questionnaire (C-BARQ) and Monash Dog–Owner Relationship Scale (MDORS) were used.

The C-BARQ, which consists of 100 items, was originally developed to investigate normal and abnormal behaviors in canine populations. In its initial formulation, it comprised 11 behavioral subscales, each representing a distinct behavioral or emotional dimension, and was designed to assess the behavioral traits of companion dogs in the United States [37]. Three additional subscales were later included [38], resulting in the current version with 14 subscales, which is widely validated in the U.S. The C-BARQ can also be administered to non-English-speaking respondents; however, it must be validated for the specific language and cultural context in which it is applied. This is necessary because significant sociocultural differences may exist among countries, for example, in the perception of the dog’s role within society and among individual owners, in the educational background of professionals, in dog-handling and training practices, and in the interpretation or acceptance of various canine behaviors. Consequently, the questionnaire has been validated and widely used in several countries. In Italy, the Italian validated version showed only partial validation for some items [39,40], and complete validation was achieved only recently [41]. Therefore, in the present study, the questionnaire was organized according to the validated Italian version developed by Broseghini et al. [41], in which 62 of the 100 questionnaire items were grouped into 13 Factors (i.e., subscales). Owners rated their dogs’ responses using a series of 5-point Likert scales, measuring either frequency (0 = never to 4 = always) or intensity (0 = no sign of the behavior to 4 = severe form of the behavior), depending on the specific item. In most cases, the Likert-scale responses indicated improvement with increasing numerical values. However, the items Q5 (Dog is slow to respond to correction or punishment), Q6 (Dog is slow to learn new tricks or tasks), and Q77 (Dog escapes or would escape from home or yard given the chance), which are included in Factor 8 in the Italian validate version, indicated worsening with higher scores. Therefore, these items were reverse scored before inclusion in the dataset. Although the Italian version of the C-BARQ has been validated, we assessed the internal reliability of the subscales using Cronbach’s *α* coefficient to ensure robustness within our specific cohort of mindfulness-interested individuals. This further validation was carried out because our participants were not randomly selected; instead, they belonged to a specific cohort (including both the mindfulness group and the control group) of individuals sharing an interest in mindfulness. We therefore reassessed internal reliability to make our results as robust as possible.

To further assess the quality of the dog–owner relationship, we administered the Monash Dog–Owner Relationship Scale (MDORS) developed by [42]. The MDORS comprises 28 items grouped into three main sections (often referred to as subscales or factors) representing distinct dimensions of the dog–human relationship. The Pet–Owner Interaction (POI) subscale reflects general activities related to the physical care of the pet and shared routines with it; the Perceived Emotional Closeness (PEC) subscale includes assessing social support, emotional bonding, psychological attachment, and companionship; and the Perceived Costs (PC) subscale encompasses items evaluating the negative aspects of pet ownership, such as the financial, social, or emotional burdens. The original MDORS was later adapted to include specific items related to cats [43], resulting in the Cat/Dog–Owner Relationship Scale (C/DORS), which integrates all MDORS items and the Cat–Owner Relationship Scale (CORS). The C/DORS is based on the same theoretical framework and has the same structure as the MDORS, but it can be applied interchangeably to both cat and dog owners. In the Italian context, a validated version of the C/DORS is available [44]; however, a formally validated Italian version of the original MDORS, developed specifically for dogs, is not yet available. Our study began before the validation of the Italian C/DORS and already included initial pilot testing; therefore, we continued with the original English version of the MDORS to maintain methodological consistency throughout the project. The items were translated by our research team and literally rendered into Italian, to preserve semantic correspondence with the original items. Responses were rated on 5-point Likert scales, depending on the item, either in terms of frequency (0 = never to 4 = very often/always) or agreement/intensity (0 = strongly disagree to 4 = strongly agree). Given the limited sample size, a full validation of the Italian version was not possible in the present study. However, a Principal Component Analysis (PCA) was conducted on all Italian translations of the questionnaire items to identify items consistent with the structure of the original English questionnaire and to remove those that were not aligned. The PCA was carried out using a Pearson correlation matrix, appropriate for data derived from Likert-type scales, with Varimax orthogonal rotation and Kaiser normalization. The adequacy of the data for PCA was assessed through the Kaiser–Meyer–Olkin (KMO) measure of sampling adequacy and Bartlett’s test of sphericity, to confirm sufficient intercorrelations among variables. Finally, the internal consistency of the resulting subscales was evaluated using Cronbach’s *α* for each extracted factor.

### 2.4. Data Organization and Statistical Analyses

The C-BARQ and MDORS databases initially contained over 400 and 200 questionnaires, respectively. Before conducting the statistical analysis, a series of preliminary procedures were performed to verify data quality and prepare the final dataset. To standardize the data, several variables were transformed: nominal responses were removed, dog age was expressed in years, and the age recorded at baseline (T0) was assigned to each subject for both time points. To ensure data integrity, a rigorous multi-step filtering process was applied. First, we excluded incomplete surveys, those with inconsistent responses (e.g., scores exceeding 4), and duplicate submissions. Second, we removed entries where the same owner evaluated multiple dogs, retaining only the first dog reported to avoid pseudo-replication. Ultimately, the final analytic sample was determined by the availability of paired data at both T0 and T1. After these exclusions, the final C-BARQ dataset included 51 paired records (T0 and T1) for the control group and 102 paired records for the mindfulness group, for a total of 306 records. For the MDORS, the final dataset comprised 27 paired questionnaires for the control group and 50 for the mindfulness group, for a total of 154 records.

The variables used for the statistical analyses were the mean values of the individual items, grouped according to their respective Factors. The first step was to assess the data distribution to determine whether it conformed to a normal distribution. Kurtosis and skewness values were within acceptable ranges, means and medians were closely aligned, and Q-Q plots were generally satisfactory. However, formal tests of normality revealed significant deviations from an ideal normal distribution, with most observations classified as non-normal by both the Shapiro–Wilk and Kolmogorov–Smirnov tests. Despite these significant results, given the other distributional indicators and the robustness of parametric tests under such conditions, we opted for a more conservative approach and employed nonparametric statistics.

A first analysis consisted of within-group pairwise comparisons using Wilcoxon signed-rank tests, conducted separately for the two conditions (Control vs. Mindfulness), to assess whether changes were specific to the Mindfulness group. Specifically, within-group Wilcoxon signed-rank tests were performed for each C-BARQ factor (F1–F13) and each MDORS section (1–3), analyzed separately for the Mindfulness and Control conditions. *p*-values were corrected using the Bonferroni method.

Subsequently, additional validation steps were applied to strengthen the findings. Specifically, internal consistency (Cronbach’s *α*) was evaluated for each relevant C-BARQ factor or MDORS section.

Baseline equivalence between groups at T0 was assessed using Mann–Whitney *U* test. This step was essential, as substantial baseline differences could influence relative changes over time.

Finally, only for those factors that showed significant differences in the uncorrected Wilcoxon pairwise tests, potential confounding effects of additional variables were examined using Generalized Linear Mixed Models (GLMMs). The models included Condition (Control vs. Mindfulness), Time (T0 vs. T1), Dog’s Sex (Male vs. Female), and Dog’s Age (as a covariate) as fixed effects, as well as Condition × Time, Condition × Dog’s Sex, and Condition × Dog’s Age interactions. Subject identity (Dog ID) was included as a random effect to account for the non-independence of repeated measures within subjects. Given the data structure and the non-normal distribution of the dependent variables, a Gamma probability distribution with a log link function was applied to the GLMMs. A compound symmetry covariance structure was assumed to account for the correlation between repeated measures within individuals across the two time points (T0 and T1). For the interpretation of model coefficients, the intercept represents the expected value of the outcome for the baseline group. In our model, the baseline categories were dummy-coded and set as the reference level (coded as 0): the Control condition at Time T0, female subjects, and the mean Age. All other coefficients represent deviations from this baseline. The GLMM approach allows for assessing whether observed changes in nonparametric tests are attributable to the intervention, while controlling for baseline imbalances and covariates.

To avoid unreliable estimates, variables such as Breed (characterized by a non-homogeneous “mixed” category and a low number of individuals per breed), along with Sex status and Owner sex (with an overwhelming majority of female owners), were excluded from the model.

All statistical analyses were performed using IBM SPSS Statistics for Windows, version 28.0 (IBM Corp., Armonk, NY, USA). The significance level for all tests was set at *p* < 0.05.

## 3. Results

The presentation of the results for both C-BARQ factors and MDORS sections follows a consistent structure. Changes between T0 (baseline) and T1 (post-intervention) are first described for each measure, following the procedure outlined in the previous section. Only factors or sections showing significant variation are discussed in detail. For these, the text reports whether the observed change occurred in the Mindfulness or Control group, and whether it was confirmed by subsequent validation analyses (Cronbach’s *α*, Mann–Whitney *U*, and GLMM results).

To maintain clarity and focus, the main text includes only numerical values referring to significant effects, while all complete non-parametric results from the Wilcoxon signed-rank tests comparing T0 and T1 within both groups are summarized in Table 1 and Table 2.

### 3.1. C-BARQ

#### 3.1.1. Factor 1—Stranger-Directed Aggression/Fear

Within-group Wilcoxon tests for Factor 1 revealed that the Control group showed no significant change over time, whereas the Mindfulness group exhibited a clear decrease in Factor 1 scores from T0 to T1 (*Z* = −6.314; *p* corrected < 0.001). The internal consistency was excellent (Cronbach’s *α* = 0.894, 12 items), and the Mann–Whitney test confirmed group homogeneity at baseline. The GLMM was statistically significant overall (*F* = 8.020; *p* < 0.001). The main effect of Time was significant (*F* = 14.325; *p* < 0.001), while the main effect of Condition was non-significant. Crucially, the Condition × Time interaction was highly significant (*F* = 19.024; *p* < 0.001), showing that the temporal change differed between the two groups. Neither Sex nor Age produced significant main effects or interactions with Condition. The Mindfulness × T1 interaction coefficient was *β* = −0.352 (95% CI [−0.511, −0.193]; *p* < 0.001), confirming a differential temporal pattern between the two groups. Specifically, considering the full model coefficients (and the Log link function), the Mindfulness group showed a decrease in Factor 1 scores over time, while the Control group exhibited a slight, non-significant increase (*β* = 0.023).

Thus, both nonparametric and GLMM analyses consistently indicate that the mindfulness intervention led to a significant reduction in stranger-directed aggression/fear over time.

#### 3.1.2. Factor 2—Dog-Directed Fear

The Wilcoxon test showed no significant difference in the Control group, whereas the Mindfulness group showed a reduction (*Z* = −2.036; *p* = 0.042); however, this effect did not remain significant after Bonferroni correction. The internal consistency was excellent (Cronbach’s *α* = 0.841, 4 items), and no baseline differences were found between groups. The overall GLMM was not statistically significant for any effects of interest, including the Condition × Time interaction (*β* = −0.061, 95% CI [−0.311, 0.188]; *p* = 0.629), indicating that changes over time did not differ between the Mindfulness and Control groups.

Taken together, the initial non-parametric finding likely reflected unsystematic variability that was no longer evident after controlling for sex and age. Therefore, no robust evidence was found that the intervention affected owner perception of dog-directed fear.

#### 3.1.3. Factor 5—Chasing

Within-group Wilcoxon tests revealed a significant decrease in the Mindfulness group (*Z* = −2.890; *p* corrected = 0.050) but not in the Control group. Internal consistency was excellent (Cronbach’s *α* = 0.823, 4 items), and no baseline differences were observed between groups. The GLMM was significant overall (*F* = 3.756; *p* = 0.001), with significant main effects of Time (*F* = 7.929; *p* = 0.005) and Sex (*F* = 12.831; *p* < 0.001). Examination of the fixed-effects coefficients showed that males scored significantly lower than females (*β* = −0.302, 95% CI [−0.555, −0.050]; *p* = 0.019), regardless of Condition. However, the Condition × Time interaction was not significant (*β* = −0.003, 95% CI [−0.113, 0.107]; *p* = 0.957). Thus, indicating that the temporal trajectories of Factor 5 did not differ between groups.

Taken together, although non-parametric analyses suggested a reduction in chasing within the Mindfulness group, this effect was not confirmed by the GLMM once covariates were controlled for. Therefore, the within-group difference should not be considered robust.

#### 3.1.4. Factor 7—Attachment/Attention Seeking

Uncorrected Wilcoxon tests suggested significant temporal changes in both groups (Control: *Z* = −2.448, *p* = 0.014; Mindfulness: *Z* = −2.246, *p* = 0.025). However, these statistical differences did not persist after Bonferroni correction for either group. Internal consistency was very good (Cronbach’s *α* = 0.735, 6 items), and no baseline differences were observed between groups. The overall GLMM was not significant, and the lack of significance for the Condition × Time interaction (*β* = 0.063, 95% CI [−0.048, 0.175]; *p* = 0.265) suggests that changes over time were similar across both groups.

Taken together, the non-parametric results were not confirmed by the GLMM, suggesting that the observed changes in attachment/attention-seeking were not robust or reliable across analyses.

#### 3.1.5. Factor 9—Energy Level

Wilcoxon tests revealed a significant decrease in the Mindfulness group (*Z* = −3.938, *p* corrected = 0.001), but not in the Control group. Internal consistency was acceptable (Cronbach’s *α* = 0.596, 3 items), and no baseline differences were detected between groups. The GLMM was statistically significant overall (*F* = 4.991, *p* < 0.001), showing main effects of Time (*F* = 16.003, *p* < 0.001) and Age (*F* = 7.484, *p* = 0.007), as well as a significant Condition × Sex interaction (*F* = 6.321, *p* = 0.013). However, the Condition × Time interaction was not significant (*β* = 0.000, 95% CI [−0.159, 0.160]; *p* = 0.997). This indicates that the reduction in Energy level was not specific to the intervention. Examination of the fixed-effect coefficients indicated that males had higher Energy level scores than females (*β* = 0.367, 95% CI [0.100, 0.633]; *p* = 0.007), although this sex difference was attenuated in the Mindfulness group (*β* = −0.433, 95% CI [−0.772, −0.094]; *p* = 0.013). In addition, scores decreased from T0 to T1 across all participants (*β* = −0.162, 95% CI [−0.291, −0.032]; *p* = 0.014), while no other effects reached statistical significance.

Overall, the absence of a significant Condition × Time interaction suggests that the reduction in Energy level observed in the Mindfulness group in the non-parametric analyses is not robust.

#### 3.1.6. Factor 11—Excitability

The uncorrected Wilcoxon test showed a decrease in the Mindfulness group (*Z* = −2.687, *p* = 0.007). However, this effect became only a statistical trend after Bonferroni correction (*p* = 0.094). Internal consistency was good (Cronbach’s *α* = 0.672, 3 items), and the two groups did not differ at baseline. The overall GLMM was statistically significant (*F* = 2.444, *p* = 0.019). Among the predictors, Time (*F* = 5.364, *p* = 0.021) and Sex (*F* = 4.296, *p* = 0.039) emerged as significant main effects. No significant effects were found for Condition, Condition × Time (*β* = −0.119, 95% CI [−0.345, 0.107]; *p* = 0.300), or any interactions involving Age, confirming that the observed changes in Excitability were not statistically robust. Inspection of the fixed-effect coefficients confirmed that males showed higher Excitability scores than females overall (*β* = 0.407, 95% CI [0.003, 0.814]; *p* = 0.050), and this sex difference was consistent across conditions.

Considering that the Bonferroni-corrected Wilcoxon results were non-significant and the GLMM revealed no Condition × Time interaction, the most parsimonious conclusion is that the observed changes in excitability were not robust.

#### 3.1.7. Other Factors

For Factors 3, 4, 6, 8, 10, 12, and 13, within-group pairwise Wilcoxon signed-rank tests indicated that scores remained stable from T0 to T1 in the Mindfulness condition, with no significant temporal changes (see Table 1). Therefore, the factors corresponding, respectively, to Owner-directed aggression, Separation-related behavior, Dog-directed aggression, Trainability, Non-social fear, Elimination problems, and Touch sensitivity showed no evidence of differences as a function of the intervention.

### 3.2. MDORS

Although the sample size did not allow a full validation of the Italian version of the MDORS, the PCA results and the exclusion of ambiguous items support its reliable use. Furthermore, the internal consistency of each component (factor), as assessed by Cronbach’s *α* coefficients (see below), confirmed the coherence of the translated items with the original questionnaire structure.

The suitability of the data for PCA was confirmed by a Kaiser–Meyer–Olkin (KMO) value of 0.706, indicating adequate sampling adequacy, and by Bartlett’s test of sphericity (*χ*^2^ = 1498.59, *p* < 0.001), confirming that the correlations among variables were sufficient to proceed with the analysis. Three principal components were extracted, and three factors were retained using Kaiser’s criterion (eigenvalues > 1). Component 1, explaining 18.5% of the variance (eigenvalue = 5.2), included items Q11–Q19 and corresponded to the Perceived Emotional Closeness section of the original English MDORS. Component 2, explaining 11.2% of the variance (eigenvalue = 3.1), included items Q20–Q28, closely matching the Perceived Costs section of the original questionnaire. Component 3, explaining 7.1% of the variance (eigenvalue = 2.0), included items Q1, Q3–Q6, Q8, and Q9, corresponding to the Dog–Owner Interaction section of the original MDORS. The cumulative variance explained by the PCA was 37%. Items Q2 and Q7 did not show factor loadings greater than 0.3 on any component, while items Q10 and Q18 showed cross-loadings greater than 0.3 on more than one component; therefore, these items were excluded from the final analysis to ensure the internal coherence of the factors.

#### Section Results

Within-group Wilcoxon signed-rank tests showed no significant changes from T0 to T1 in the Mindfulness condition across all three MDORS sections: Dog–Owner Interaction, Perceived Emotional Closeness, and Perceived Costs, indicating temporal stability of these domains. The Control group also remained stable, except for Section 2 (Perceived Emotional Closeness), which showed a significant decrease (*Z* = −2.650; *p* corrected = 0.024). Therefore, this factor does not meet the test–retest reliability criterion in the current sample, whereas the Mindfulness group maintained stability over time.

## 4. Discussion

In this study, we observed significant changes in several factors of the Canine Behavioral Assessment and Research Questionnaire (C-BARQ) following a mindfulness intervention for dog owners.

Our analysis revealed several specific indicators of a shift in owner perception. Five factors showed consistent, improvement-oriented changes. However, four of these should be interpreted with caution, as the findings from initial non-parametric tests were not supported by the Generalized Linear Mixed Models (GLMMs). These tentative findings suggest that owners who underwent mindfulness training tended to perceive their dogs as being less fearful of other dogs, exhibiting reduced predatory chasing tendencies, and possessing lower energy levels (i.e., being calmer and less excitable) compared to their perceptions before the mindfulness course. One factor, however, proved particularly robust, as this effect was confirmed by the GLMMs: a perceived reduction in the dogs’ fear of and aggression towards strangers.

While these changes are statistically robust in some instances, we posit that they are unlikely to reflect a meaningful, fundamental shift in the dogs’ behavioral tendencies over a relatively short 8-week intervention period, especially in the absence of any specific training or treatment directed at the animals. Although the possibility of indirect change cannot be entirely excluded, for instance, modifications in the owner’s behavior or household dynamics could influence the dog [30]. Considering the well-established emotional and physiological contagion between humans and dogs [45,46,47], such modifications could potentially decrease the dog’s arousal in the presence of strangers, possibly leading to less fear or aggression toward the strangers. Nevertheless, this remains a hypothesis that requires validation through direct behavioral and physiological research. However, given the absence of objective, non-invasive behavioral or physiological measures in dogs, this hypothesis cannot be directly tested here. A more accurate conclusion, supported by our owner-reported data, is that the results reflect the psychological effects of mindfulness on the owners themselves. It is more probable that the increased calmness and reduced anxiety often associated with mindfulness practice led owners to perceive their dog’s negative behaviors as less pronounced or problematic. This interpretation is consistent with established knowledge that owners’ perceptions of their dogs are highly susceptible to influence [48]. In this context, it is equally important to consider the factors for which the owners’ perception did not show significant changes, such as owner-directed aggression, separation-related behavior, dog-directed aggression, trainability, non-social fear, elimination problems, and touch sensitivity. The stability of these scores suggests that certain behavioral domains, or the owner’s assessment of them, may be more resistant to the psychological shifts induced by a short-term mindfulness intervention. While the relatively small sample size of this pilot study may have limited the statistical power to detect subtle changes in these categories, it is worth noting that effects requiring very large cohorts to reach significance often possess low practical magnitude. Therefore, our results prioritize the most robust perceptual shifts, such as the reduction in perceived stranger-directed aggression, where the impact was strong enough to emerge clearly despite the inherent variability of the sample. Although direct behavioral observations are lacking, the literature suggests a plausible indirect pathway: practicing mindfulness may reduce owners’ anxiety [49] and promote more prosocial or open attitudes [50,51], which in turn could influence how owners perceive and report their dogs’ behavior.

Conversely, the Monash Dog Owner Relationship Scale (MDORS), a questionnaire specifically developed to study the owner-dog attachment relationship, did not detect any significant effects of the intervention. Similarly, the attachment-related factor (Factor 7) of the C-BARQ yielded unreliable results due to its lack of established test–retest reliability. Our results are in line with a previous study on mindful practice with dogs, which found no change over time in a quantitative sense after two 6-week interventions [49]. This pattern of results leads to a key conclusion: while the core emotional aspects of the owner-dog bond, as measured by attachment-specific tools, remained stable, the owner’s perception of the dog’s day-to-day behavioral tendencies was more dynamic and positively influenced by the mindfulness practice. This raises an important consideration regarding the constructs being measured. While the MDORS investigates aspects of the relationship assumed to be stable over short periods, our findings suggest that owner perception itself can be a more fluid variable. A possible alternative explanation is that the study participants selected already had high levels of Factors 1 and 2 and a low level of Factor 3 at T0. The absence of change in Factors 1 and 2 may reflect a ceiling effect, whereas the lack of decrease in Factor 3 could indicate a floor effect. A relevant limitation concerns the use of the MDORS. Although the translation of the scale followed standard procedures and the subsequent item reduction via PCA represents a defensible exploratory approach, this procedure cannot be considered equivalent to the use of a fully validated Italian version of the instrument. In addition, the smaller sample size available for the MDORS analyses limits the interpretability of the null findings, making it difficult to disentangle a true absence of effects from limited statistical power or measurement constraints. These factors may have contributed to the apparent stability of relationship domains over time. Future studies should therefore employ an appropriately validated Italian instrument specifically designed to assess the owner–dog relationship, as suggested by existing literature, to more robustly evaluate potential changes in attachment-related constructs.

The central novelty of our findings lies in the demonstration that the potential psychological shifts associated with owner mindfulness, such as a possible reduction in anxiety or heightened calmness, are reflected in how owners report their dogs’ behaviors through C-BARQ factors. While these internal psychological states were not directly measured in the present study, the shift in owner perception suggests that the dog may act as a “sensor” for the owner’s subjective experience of their immediate environment. Our overall results indicate a generalized enhancement in perception, with no concurrent worsening in any assessed domain. This suggests that the influence of mindfulness on owner perception is not uniform but is specifically targeted towards the interpretation of certain canine behaviors. The core insight is not solely about the assumed benefits of mindfulness for the owner, but rather that the C-BARQ acts as a sensitive, ecologically valid instrument capable of measuring the potential transmission of the owner’s internal state into their subjective perception of their dog’s behavior. This interpretation aligns seamlessly with the extensive literature on the broad psychological benefits of mindfulness [4,10,11,12,13,52,53,54,55]. At a cognitive and emotional level, it has been shown to enhance functions like attention and reaction time [15], increase self-acceptance and self-compassion, and reduce negative emotions and self-evaluations [16], all of which contribute to lower anxiety and stress [14]. Our study provides a novel, ecologically valid example of this phenomenon, demonstrating that these internal shifts in the owner’s well-being can manifest as altered, more positive perceptions of their companion animal’s behavior.

However, if this change primarily reflects a shift in owner perception rather than an actual change in the dog’s behavior, it would warrant careful consideration with respect to animal welfare and public safety. While mindfulness may help owners manage the emotional burden of canine reactivity, there is a theoretical risk that an increased state of calmness could lead to an underestimation or misinterpretation of the dog’s actual distress or signals of impending aggression. If mindfulness training results in decreased vigilance or an overly optimistic appraisal of the dog’s behavior, it could inadvertently compromise the dog’s welfare or increase the risk of dangerous incidents with strangers. This highlights the need for future research to determine whether these perceptual changes correspond to accurate interpretations of canine body language. A valuable follow-up study could involve assessing whether mindfulness-trained owners can accurately identify signs of fear or aggression in standardized video observations compared to untrained controls.

While this represents the first study to explore the impact of owner mindfulness on canine behavior using the C-BARQ, some methodological limitations must be acknowledged. The reliance solely on owner-reported measures during the intervention fundamentally prevents us from distinguishing between an actual change in the dog’s behavior, a change in the owner’s perception of that behavior, or both.

A further limitation concerns the gender imbalance of the sample, which is overwhelmingly composed of women. This imbalance in the sex ratio among participating owners prevents us from adequately generalizing the results to both genders. Although this finding is common in self-reported questionnaire studies concerning human–animal interaction, where women are often the primary caregivers and handlers of the dog [18,49], it represents a potential gender bias. Since owner sex was excluded from the GLMMs due to the highly uneven distribution, we could not analyze direct interactions between owner sex and the intervention. It is crucial to discuss that the obtained results, particularly the perception of reduced aggression toward strangers, might be specific to a predominantly female cohort. Specifically, prior research demonstrates that women, typically report higher levels of positive attitudes and attachment towards companion animals [56]. This differential may increase their sensitivity and propensity to report positive behavioral changes following an intervention. Therefore, the generalizability of these results to the male dog-owner population is limited, and future studies should aim for more balanced sampling to confirm the effect of mindfulness regardless of the owner’s gender, as well as to examine the potential influence of breed.

## 5. Conclusions

The results of the current study indicate that an owner’s mindfulness practice may influence their perceptions of their dogs’ behaviors (C-BARQ), while not affecting the relationship (MDORS). Although the attachment bond, as measured by the MDORS, remained steady, owners’ daily perceptions of their dogs’ behavior, most notably stranger-directed aggression, were more influenced by the intervention’s effects. These findings suggest that mindfulness can enhance the owner’s subjective well-being, which in turn could positively impact how they interpret their environment and their pet. The key point to highlight is that, across all the parameters analyzed, owners’ evaluations never showed any worsening, either in the behavioral measures or in the relationship with their dogs. Future long-term studies utilizing more balanced samples and extended follow-up periods must integrate direct behavioral and physiological measures in dogs. This approach will be necessary to determine whether these perceptual shifts extend to deeper relational aspects and represent actual changes in canine behavior.

## Figures and Tables

**Table 1:** Results of within-group Wilcoxon signed-rank test comparing pre-intervention (T0) and post-intervention (T1) scores for each C-BARQ behavioral factor (F1–F13) in the Control (n = 51) and Mindfulness (n = 102) groups. Values are presented as medians (Mdn). *p*-values were adjusted using the Bonferroni correction. Significant differences are indicated as follows: * *p* < 0.05, ** *p* < 0.01, *** *p* < 0.001. *Z* = standardized test statistic; *p* = uncorrected two-tailed *p* value; *p* (Bonf.) = Bonferroni-corrected two-tailed *p* value.

	C-BARQ Control					C-BARQ Mindfulness				
Factor	T0 (Mdn)	T1 (Mdn)	*Z*	*p*	*p* (Bonf.)	T0 (Mdn)	T1 (Mdn)	*Z*	*p*	*p* (Bonf.)
F1_StrangerAggFear	0.75	0.67	−0.094	0.925	1.000	0.67	0.38	−6.314	<0.001 ***	<0.001 ***
F2_DogFear	0.50	0.25	−0.423	0.672	1.000	0.75	0.67	−2.036	0.042 *	0.543
F3_OwnerAgg	0.00	0.00	−0.697	0.486	1.000	0.00	0.00	−0.080	0.936	1.000
F4_Separation	0.50	0.50	−0.952	0.341	1.000	0.33	0.33	−0.185	0.853	1.000
F5_Chasing	2.67	2.50	−1.876	0.061	0.788	2.50	2.29	−2.890	0.004 **	0.050 *
F6_DogAgg	0.88	0.75	−0.896	0.370	1.000	1.00	1.00	−0.357	0.721	1.000
F7_Attach	2.17	2.00	−2.448	0.014 *	0.187	2.00	1.83	−2.246	0.025 *	0.321
F8_Trainability	3.00	3.00	−0.789	0.430	1.000	3.00	3.00	−1.588	0.112	1.000
F9_Energy	1.33	1.33	−1.900	0.057	0.746	1.33	1.00	−3.938	<0.001 ***	0.001 **
F10_NonSocialFear	1.00	1.00	−0.851	0.395	1.000	1.00	1.00	−1.046	0.296	1.000
F11_Excitability	1.67	1.67	−0.622	0.534	1.000	1.50	1.33	−2.687	0.007 **	0.094
F12_Elimination	0.00	0.00	−2.376	0.017 *	0.227	0.00	0.00	−0.764	0.445	1.000
F13_TouchSens	0.50	0.50	−0.507	0.612	1.000	0.33	0.33	−0.397	0.692	1.000

**Table 2:** Results of within-group Wilcoxon signed-rank tests comparing pre-intervention (T0) and post-intervention (T1) scores for the MDORS sections in the Control (n = 27) and Mindfulness (n = 50) groups. Section 1 = Dog–Owner Interaction; Section 2 = Perceived Emotional Closeness; Section 3 = Perceived Costs. Values are presented as medians (Mdn). *p*-values were adjusted using the Bonferroni correction. Significant differences (*p* < 0.05) are indicated in bold: * *p* < 0.05, ** *p* < 0.01. *Z* = standardized test statistic; *p* = uncorrected two-tailed *p* value; *p* (Bonf.) = Bonferroni-corrected two-tailed *p* value.

	MDORS Control					MDORS Mindfulness				
Section	T0 (Mdn)	T1 (Mdn)	*Z*	*p*	*p* (Bonf.)	T0 (Mdn)	T1 (Mdn)	*Z*	*p*	*p* (Bonf.)
Section 1	3.43	3.43	−1.447	0.148	0.444	3.29	3.29	−0.630	0.529	1.000
Section 2	3.50	3.25	−2.650	0.008 **	0.024 *	3.38	3.38	−1.606	0.108	0.325
Section 3	0.33	0.56	−0.658	0.510	1.000	0.44	0.44	−0.433	0.665	1.000

## Data Availability

The data presented in this study are available on request from the corresponding author.

## References

[1] Nnanavamsa, Krishnasamy P. (2014). Buddha’s Philosophical Interpretation of Eightfold Path. Indian Streams Res. J..

[2] Schmidt S. (2011). Mindfulness in East and West—Is It the Same?. Neuroscience, Consciousness and Spirituality.

[3] Baer R.A. (2003). Mindfulness Training as a Clinical Intervention: A Conceptual and Empirical Review. Clin. Psychol. Sci. Pract..

[4] Kabat-Zinn J. (2003). Mindfulness-Based Interventions in Context: Past, Present, and Future. Clin. Psychol. Sci. Pract..

[5] Klatt M.D., Buckworth J., Malarkey W.B. (2009). Effects of Low-Dose Mindfulness-Based Stress Reduction (MBSR-Ld) on Working Adults. Health Educ. Behav..

[6] Kabat-Zinn J. (1990). Full Catastrophe Living: Using the Wisdom of Your Body and Mind to Face Stress, Pain, and Illness.

[7] Segal Z.V., Anderson A.K., Gulamani T., Williams L.-A.D., Desormeau P., Ferguson A., Walsh K., Farb N.A.S. (2019). Practice of Therapy Acquired Regulatory Skills and Depressive Relapse/Recurrence Prophylaxis Following Cognitive Therapy or Mindfulness Based Cognitive Therapy. J. Consult. Clin. Psychol..

[8] Kropp A., Sedlmeier P. (2019). What Makes Mindfulness-Based Interventions Effective? An Examination of Common Components. Mindfulness.

[9] Bishop S.R., Lau M., Shapiro S., Carlson L., Anderson N.D., Carmody J., Segal Z.V., Abbey S., Speca M., Velting D. (2004). Mindfulness: A Proposed Operational Definition. Clin. Psychol. Sci. Pract..

[10] Call D., Miron L., Orcutt H. (2014). Effectiveness of Brief Mindfulness Techniques in Reducing Symptoms of Anxiety and Stress. Mindfulness.

[11] Colgan D.D., Christopher M., Michael P., Wahbeh H. (2016). The Body Scan and Mindful Breathing Among Veterans with PTSD: Type of Intervention Moderates the Relationship Between Changes in Mindfulness and Post-Treatment Depression. Mindfulness.

[12] Corbett C., Egan J., Pilch M. (2019). A Randomised Comparison of Two ‘Stress Control’ Programmes: Progressive Muscle Relaxation versus Mindfulness Body Scan. Ment. Health Prev..

[13] Kabat-Zinn J. (2019). You Can’t Get There from Here. Mindfulness.

[14] Kogan L.R., Bussolari C. (2021). Exploring the Potential Impact of a Virtual Body Scan Meditation Exercise Conducted with Pet Dogs on Recipients and Facilitators. Front. Psychol..

[15] Adhikari K., Kothari F., Khadka A. (2018). The Effect of Short-Term Training of Vipassana’s Body-Scan on Select Cognitive Functions. Psychol. Stud..

[16] Schultchen D., Messner M., Karabatsiakis A., Schillings C., Pollatos O. (2019). Effects of an 8-Week Body Scan Intervention on Individually Perceived Psychological Stress and Related Steroid Hormones in Hair. Mindfulness.

[17] Goyal M., Singh S., Sibinga E.M.S., Gould N.F., Rowland-Seymour A., Sharma R., Berger Z., Sleicher D., Maron D.D., Shihab H.M. (2014). Meditation Programs for Psychological Stress and Well-Being: A Systematic Review and Meta-Analysis. JAMA Intern. Med..

[18] Hawkins R.D., Hawkins E.L., Tip L. (2021). “I Can’t Give Up When I Have Them to Care for”: People’s Experiences of Pets and Their Mental Health. Anthrozoos.

[19] Gee N.R., Rodriguez K.E., Fine A.H., Trammell J.P. (2021). Dogs Supporting Human Health and Well-Being: A Biopsychosocial Approach. Front. Vet. Sci..

[20] Gee N.R., Mueller M.K. (2019). A Systematic Review of Research on Pet Ownership and Animal Interactions among Older Adults. Anthrozoos.

[21] Wells D.L. (2004). The Facilitation of Social Interactions by Domestic Dogs. Anthrozoos.

[22] Guéguen N., Ciccotti S. (2008). Domestic Dogs as Facilitators in Social Interaction: An Evaluation of Helping and Courtship Behaviors. Anthrozoos.

[23] Henry C.L., Crowley S.L. (2015). The Psychological and Physiological Effects of Using a Therapy Dog in Mindfulness Training. Anthrozoos.

[24] Navarro-Haro M.V., López-del-Hoyo Y., Campos D., Linehan M.M., Hoffman H.G., García-Palacios A., Modrego-Alarcón M., Borao L., García-Campayo J. (2017). Meditation Experts Try Virtual Reality Mindfulness: A Pilot Study Evaluation of the Feasibility and Acceptability of Virtual Reality to Facilitate Mindfulness Practice in People Attending a Mindfulness Conference. PLoS ONE.

[25] Chandrasiri A., Collett J., Fassbender E., De Foe A. (2020). A Virtual Reality Approach to Mindfulness Skills Training. Virtual Real..

[26] Seabrook E., Kelly R., Foley F., Theiler S., Thomas N., Wadley G., Nedeljkovic M. (2020). Understanding How Virtual Reality Can Support Mindfulness Practice: Mixed Methods Study. J. Med. Internet Res..

[27] Amiot C.E., Quervel-Chaumette M., Gagné C., Bastian B. (2025). An Experimental Study Focusing on Mindfulness to Capture How Our Contacts with Dogs Can Promote Human Well-Being. Sci. Rep..

[28] Pérez-Manrique A., Gomila A. (2022). Emotional Contagion in Nonhuman Animals: A Review. WIREs Cogn. Sci..

[29] González-Ramírez M.T., Vanegas-Farfano M., Landero-Hernández R. (2018). Differences in Stress and Happiness between Owners Who Perceive Their Dogs as Well Behaved or Poorly Behaved When They Are Left Alone. J. Vet. Behav..

[30] Dodman N.H., Brown D.C., Serpell J.A. (2018). Associations between Owner Personality and Psychological Status and the Prevalence of Canine Behavior Problems. PLoS ONE.

[31] Djernis D., Lerstrup I., Poulsen D., Stigsdotter U., Dahlgaard J., O’toole M. (2019). A Systematic Review and Meta-Analysis of Nature-Based Mindfulness: Effects of Moving Mindfulness Training into an Outdoor Natural Setting. Int. J. Environ. Res. Public Health.

[32] Choe E.Y., Jorgensen A., Sheffield D. (2020). Does a Natural Environment Enhance the Effectiveness of Mindfulness-Based Stress Reduction (MBSR)? Examining the Mental Health and Wellbeing, and Nature Connectedness Benefits. Landsc. Urban Plan..

[33] Huynh T., Torquati J.C. (2019). Examining Connection to Nature and Mindfulness at Promoting Psychological Well-Being. J. Environ. Psychol..

[34] Bragg R., Atkins G. (2016). A Review of Nature-Based Interventions for Mental Health Care. Nat. Engl. Comm. Rep..

[35] Burgon H.L. (2013). Horses, Mindfulness and the Natural Environment. Equine-Assisted Therapy and Learning with At-Risk Young People.

[36] Burger T., Potgieter J.C., Nell W. (2024). Methods and Environmental Conditions Typical of Nature-Based Mindfulness Practice: A Scoping Review. Mindfulness.

[37] Hsu Y., Serpell J.A. (2003). Development and Validation of a Questionnaire for Measuring Behavior and Temperament Traits in Pet Dogs. J. Am. Vet. Med. Assoc..

[38] Duffy D.L., Serpell J.A. (2012). Predictive Validity of a Method for Evaluating Temperament in Young Guide and Service Dogs. Appl. Anim. Behav. Sci..

[39] Marshall-Pescini S., Valsecchi P., Petak I., Accorsi P.A., Previde E.P. (2008). Does Training Make You Smarter? The Effects of Training on Dogs’ Performance (*Canis familiaris*) in a Problem Solving Task. Behav. Process..

[40] Dalla Villa P., Barnard S., Di Nardo A., Iannetti L., Vulpiani M.P., Trentini R., Serpell J.A., Siracusa C. (2017). Validation of the Socially Acceptable Behaviour (SAB) Test in a Central-Italy Pet Dog Population. Vet. Ital..

[41] Broseghini A., Guérineau C., Lõoke M., Mariti C., Serpell J., Marinelli L., Mongillo P. (2023). Canine Behavioral Assessment and Research Questionnaire (C-BARQ): Validation of the Italian Translation. Animals.

[42] Dwyer F., Bennett P.C., Coleman G.J. (2006). Development of the Monash Dog Owner Relationship Scale (MDORS). Anthrozoos.

[43] Howell T.J., Bowen J., Fatjó J., Calvo P., Holloway A., Bennett P.C. (2017). Development of the Cat-Owner Relationship Scale (CORS). Behav. Process..

[44] Riggio G., Piotti P., Diverio S., Borrelli C., Di Iacovo F., Gazzano A., Howell T.J., Pirrone F., Mariti C. (2021). The Dog–Owner Relationship: Refinement and Validation of the Italian c/Dors for Dog Owners and Correlation with the Laps. Animals.

[45] Sundman A.S., Van Poucke E., Holm A.C.S., Faresjö Å., Theodorsson E., Jensen P., Roth L.S.V. (2019). Long-Term Stress Levels Are Synchronized in Dogs and Their Owners. Sci. Rep..

[46] Aria M., Alterisio A., Scandurra A., Pinelli C., D’Aniello B. (2021). The Scholar’s Best Friend: Research Trends in Dog Cognitive and Behavioral Studies. Anim. Cogn..

[47] D’Aniello B., Semin G.R., Alterisio A., Aria M., Scandurra A. (2018). Interspecies Transmission of Emotional Information via Chemosignals: From Humans to Dogs (*Canis lupus familiaris*). Anim. Cogn..

[48] Amici F., Waterman J., Kellermann C.M., Karimullah K., Bräuer J. (2019). The Ability to Recognize Dog Emotions Depends on the Cultural Milieu in Which We Grow Up. Sci. Rep..

[49] Oliva J.L., Green T.R., Yeung P., Prato-Previde E. (2021). Dog Tales: Mindful Dog Interactions Evoke Similar Experiences to Dog Assisted Mindfulness Meditations. Animals.

[50] Blackwell C., Swickert R. (2025). Mindfulness and Trust: Survey Evidence on the Relationship Between Mindfulness Practice, Trait Mindfulness, and General Social Trust. Soc. Sci. Q..

[51] Fuochi G., Boin J., Lucarini A., Voci A. (2023). A Mindful Path Toward Prejudice Reduction: Key Mindfulness Facets and Mediators for Promoting Positive Intergroup Relations. Mindfulness.

[52] Galante J., Friedrich C., Dawson A.F., Modrego-Alarcón M., Gebbing P., Delgado-Suárez I., Gupta R., Dean L., Dalgleish T., White I.R. (2021). Mindfulness-Based Programmes for Mental Health Promotion in Adults in Nonclinical Settings: A Systematic Review and Meta-Analysis of Randomised Controlled Trials. PLoS Med..

[53] Hilton L., Hempel S., Ewing B.A., Apaydin E., Xenakis L., Newberry S., Colaiaco B., Maher A.R., Shanman R.M., Sorbero M.E. (2017). Mindfulness Meditation for Chronic Pain: Systematic Review and Meta-Analysis. Ann. Behav. Med..

[54] Beccia A.L., Dunlap C., Hanes D.A., Courneene B.J., Zwickey H.L. (2018). Mindfulness-Based Eating Disorder Prevention Programs: A Systematic Review and Meta-Analysis. Ment. Health Prev..

[55] Taylor J., McLean L., Korner A., Stratton E., Glozier N. (2020). Mindfulness and Yoga for Psychological Trauma: Systematic Review and Meta-Analysis. J. Trauma Dissociation.

[56] Herzog H. (2011). The Impact of Pets on Human Health and Psychological Well-Being: Fact, Fiction, or Hypothesis?. Curr. Dir. Psychol. Sci..

